# Identifying vulnerable plaques: A 3D carotid plaque radiomics model based on HRMRI

**DOI:** 10.3389/fneur.2023.1050899

**Published:** 2023-01-26

**Authors:** Xun Zhang, Zhaohui Hua, Rui Chen, Zhouyang Jiao, Jintao Shan, Chong Li, Zhen Li

**Affiliations:** ^1^Department of Endovascular Surgery, First Affiliated Hospital of Zhengzhou University, Zhengzhou, Henan, China; ^2^Department of Magnetic Resonance Imaging, First Affiliated Hospital of Zhengzhou University, Zhengzhou, Henan, China; ^3^Division of Vascular Surgery, New York University Medical Center, New York, NY, United States

**Keywords:** carotid atherosclerosis (AS), radiomics, 3D reconstruction, vulnerable plaque, high-resolution magnetic resonance imaging, stroke

## Abstract

**Background:**

Identification of vulnerable carotid plaque is important for the treatment and prevention of stroke. In previous studies, plaque vulnerability was assessed qualitatively. We aimed to develop a 3D carotid plaque radiomics model based on high-resolution magnetic resonance imaging (HRMRI) to quantitatively identify vulnerable plaques.

**Methods:**

Ninety patients with carotid atherosclerosis who underwent HRMRI were randomized into training and test cohorts. Using the radiological characteristics of carotid plaques, a traditional model was constructed. A 3D carotid plaque radiomics model was constructed using the radiomics features of 3D T_1_-SPACE and its contrast-enhanced sequences. A combined model was constructed using radiological and radiomics characteristics. Nomogram was generated based on the combined models, and ROC curves were utilized to assess the performance of each model.

**Results:**

48 patients (53.33%) were symptomatic and 42 (46.67%) were asymptomatic. The traditional model was constructed using intraplaque hemorrhage, plaque enhancement, wall remodeling pattern, and lumen stenosis, and it provided an area under the curve (AUC) of 0.816 vs. 0.778 in the training and testing sets. In the two cohorts, the 3D carotid plaque radiomics model and the combined model had an AUC of 0.915 vs. 0.835 and 0.957 vs. 0.864, respectively. In the training set, both the radiomics model and the combination model outperformed the traditional model, but there was no significant difference between the radiomics model and the combined model.

**Conclusions:**

HRMRI-based 3D carotid radiomics models can improve the precision of detecting vulnerable carotid plaques, consequently improving risk classification and clinical decision-making in patients with carotid stenosis.

## Introduction

The most common type of cerebrovascular disease is ischemic stroke and 15–20% of these are caused by carotid artery stenosis (1, 2). By 2020, the global prevalence of carotid plaque among people between the ages of 30 and 79 years was about 20%, and 816 million patients were reported with carotid stenosis (3). The current guidelines account for the patient's clinical presentation and the degree of carotid stenosis to determine a need for surgical intervention (4). However, as radiographic methods have limited accuracy in identifying the degree of histological stenosis in carotid arteries, imaging screening is not recommended in the general population (5). Numerous studies have demonstrated that it is essential to identify vulnerable plaques by analyzing their composition, as it has implications for a patient's clinical presentation and future cerebral ischemic events (6). Recently, magnetic resonance imaging (MRI) has been effective for identifying intraplaque hemorrhage (IPH) and lipid-rich necrotic cores (LRNC), but an atherosclerotic plaque is complex in structure and necessitates specialized knowledge of MRI for assessing plaque composition (7). Even the most advanced high-resolution magnetic resonance imaging (HRMRI) can only demonstrate the qualitative and subjective identification of lesions' structural characteristics. In the majority of previous studies, plaque composition was qualitatively quantified (8).

Radiomics is a computational technique for extracting and statistically assessing vast quantities of image texture information from medical images (9). It has been proven to be effective in oncology owing to its numerous applications in the diagnosis, grading, and staging of cancer, evaluating therapeutic efficacy, and predicting clinical outcomes (10). Computed tomography (CT) or ultrasound-based radiomics models reportedly have a potential clinical application for diagnosing carotid plaque vulnerability, whereas HRMRI-based radiomics models have received less attention and have extracted radiomics features of the carotid plaque only at the most stenotic level (11–13). In addition, it has been shown that radiomics models based on 3D HRMRI can accurately identify high-risk intracranial plaques (14). HRMRI has clear advantages in assessing vessel wall composition, and its three-dimensional T_1_ weighted sampling perfection with application-optimized contrasts by using different flip angle evolutions (3D T_1_-SPACE) sequence can provide three-dimensional, large-area, high-spatial-resolution imaging of the arterial wall (15). This study aims to develop a 3D HRMRI-based carotid radiomics model to investigate the importance of radiomics in analyzing carotid atherosclerotic plaques, improving the accuracy of vulnerable plaque identification and furthering data on the management of asymptomatic carotid stenosis.

## Materials and methods

### Study population

In this retrospective study, patients with carotid artery stenosis who underwent HRMRI at the First Affiliated Hospital of Zhengzhou University between June 2021 and June 2022 were enrolled. All patients (*N* = 90) had 50% carotid stenosis. Patients (*n* = 42) who were noted with carotid stenosis upon physical examination but had never experienced a transient ischemic attack (TIA) or stroke in the past 6 months and any radiological finding of cerebral infarction were grouped into the asymptomatic group. In symptomatic atherosclerosis, plaque enhancement subsides over time after an ischemic stroke (16). In addition, to approximate the state of carotid plaque at the time of rupture, we included patients in the symptomatic plaque group whose MRI demonstrated the presence of an acute phase (<4 weeks) of cerebral infarction in the ipsilateral blood supply area of carotid stenosis. All patients with symptomatic carotid stenosis met the Trial of Org 101072 in Acute Stroke Treatment (TOAST) criteria for “atherosclerotic TIA/ischemic stroke” before their inclusion in the study (17). Patients were excluded if they had subacute or old infarct foci on MRI, combined intracranial vascular lesions such as severe stenosis or occlusion of the anterior or middle cerebral artery, Moyamoya disease, poor HRMRI imaging, or absence of 3D T_1_- SPACE sequences.

The clinical characteristics of the patients were gathered for both groups (Table 1), and they were then randomly split into a training set (*n* = 63) and a testing set (*n* = 27) in a 7:3 ratio. The training set was used to build the radiomics model whereas the testing set was utilized to validate the model's diagnostic performance. This study protocol was reviewed and approved by our institution's ethics committee, and all patients provided informed permission.

**Table 1:** Clinical and radiological characteristics of patients.

**Parameters**	**Asymptomatic (*n* = 42)**	**Symptomatic (*n* = 48)**	***p***
Age (year)	58.43 ± 10.62	59.27 ± 11.16	0.716
Sex (female)	19 (45.23)	13 (27.08)	0.082
BMI (kg/m^2^)	24.72 ± 2.83	24.78 ± 2.66	0.649
**Clinical features**			
CAD	4 (9.52)	9 (18.75)	0.245
Hypertension	22 (52.38)	31 (64.58)	0.286
Diabetes	9 (21.43)	19 (39.58)	0.072
Current smoking	10 (23.81)	18 (37.50)	0.290
**Current medications**			
Aspirin	9 (21.43)	6 (12.50)	0.274
Clopidogrel	5 (11.90)	3 (6.25)	0.465
Statin	9 (21.43)	4 (8.33)	0.131
**Serum lipid (mmol/L)**			
Total cholesterol	3.77 ± 0.93	3.63 ± 1.07	0.521
Triglycerides	1.64 ± 1.05	1.58 ± 0.92	0.783
HDL-C	1.08 ± 0.36	1.03 ± 0.31	0.508
LDL-C	2.14 ± 0.78	2.01 ± 0.86	0.453
**Plaque characteristics**			
IPH	4 (9.52)	14 (29.17)	0.026
RMP (Positive)	6 (14.29)	15 (31.25)	0.063
PE (Apparent)	3 (7.14)	6 (12.50)	0.404
Degree of luminal stenosis	67.49 ± 9.71	76.09 ± 5.21	0.001

Data were expressed as means ± SD for continuous variables and number (percentage) for dichotomous variables. CAD, coronary artery disease; HDL-C, high-density lipoprotein-cholesterol; LDL-C, low-density lipoprotein-cholesterol; IPH, Intraplaque hemorrhage; RMP, Remodeling pattern; PE, Plaque enhancement.

### MRI acquisition

All patients were scanned with a 3T MRI (Magnetom Verio, Siemens Healthineers) with a 64-channel coil. Subjects were reminded to avoid swallowing and neck movements before the examination. The 3D T_1_- SPACE and its contrast-enhanced sequence (3D T_1_-SPACE-CE) scan were performed in the oblique coronal position. The following parameters were applied to these image sequences for diffusion-weighted imaging: 4,000 ms Repetition Time (TR), 60 ms echo time (TE), the field of view (FOV) 200×200 mm, matrix size 150 ×150, and slice thickness 2 mm; and for 3D T_1_-SPACE: TR 900 ms, TE 15 ms, FOV 200 × 224 mm, and slice thickness 0.63 mm. Before the acquisition of the 3D T_1_-SPACE-CE sequence, 0.1 mL/kg of a Gadopentetate Dimeglumine was administered to the patient.

### Image analysis and segmentation

A radiologist with 4 years of experience in vascular wall imaging performed conventional measures and segmentation while blinded to the clinical information. On 3D T_1_-SPACE and its enhanced sequence, the HRMRI characteristics, including intraplaque hemorrhage (IPH), plaque enhancement (PE), wall remodeling pattern, and lumen stenosis, were manually measured. The related methods to measure were as follows: (1) IPH: On T_1_WI, the signal intensity of plaque exceeded 150% of normal brain parenchyma.; (2) PE was classified as mild and apparent enhancement based on a comparison of plaque enhancement with the pituitary funnel stalk; (3) The wall remodeling pattern was divided into positive and negative patterns according to the ratio of the vessel area at the site of maximum luminal stenosis to the reference vessel area; (4) Diameter stenosis rate = [1 – narrow lumen area/reference lumen area] × 100% (18).

**Figure 1:**
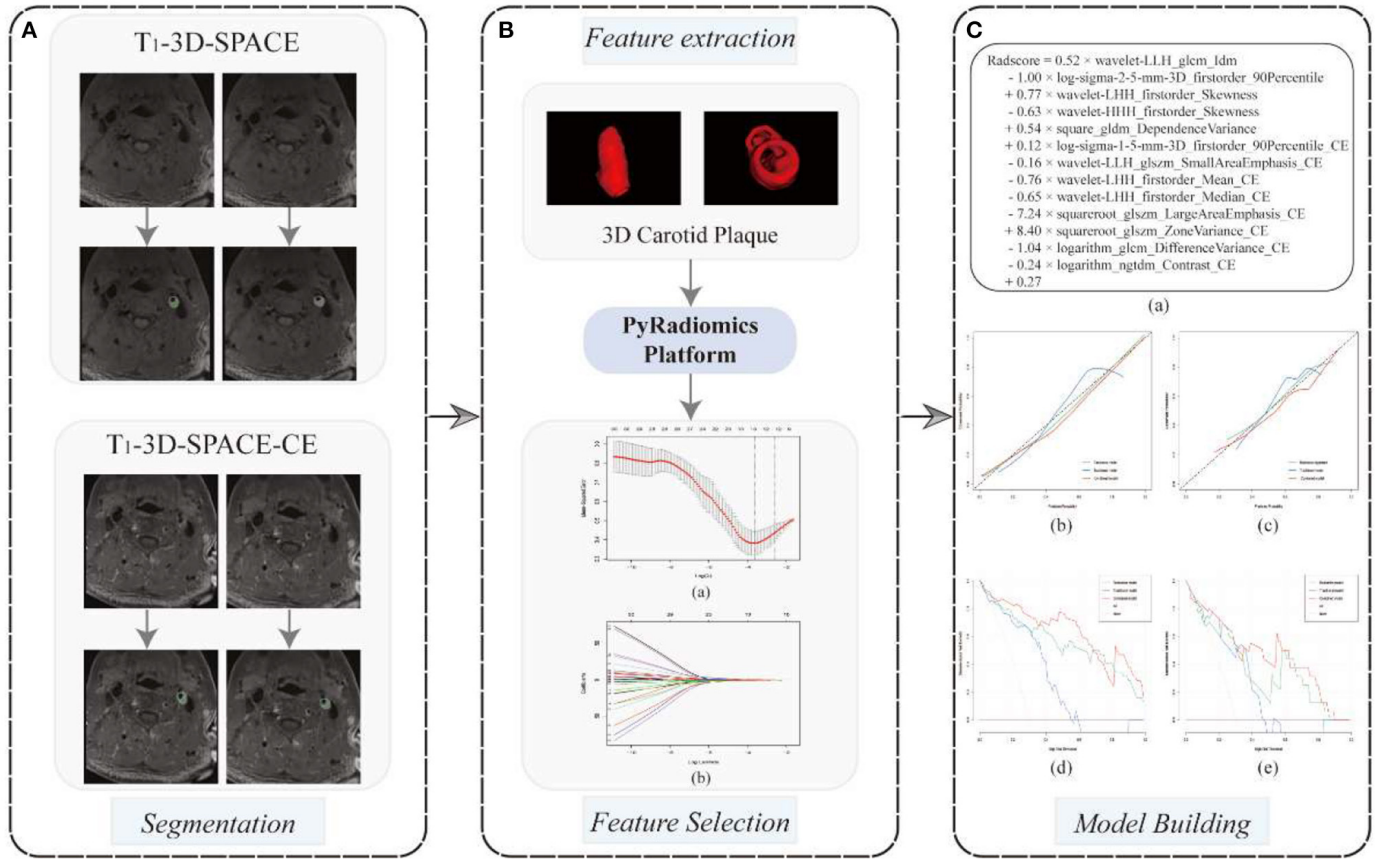
A flowchart of radiomics model development. **(A)** 3D carotid plaque segmentation in 3D T_1_-SPACE and 3D T_1_-SPACE- CE sequences, respectively; **(B)** PyRadiomics-based radiomics feature extraction, 13radiomics features were screened at the minimum mean square error of the LASSO regression and used to build radiomics models (a, b); **(C)** The formulation of radiomics signature (a); The calibration curves (b, c) and decision curves analysis (d, e) for the training and testing cohorts were employed to evaluate the traditional model, the radiomics model and the combined model, respectively.

Utilizing the free program 3D Slicer (version 4.13.0, www.slicer.org), plaque segmentation was carried out for radiomics investigation. After identifying all slices containing plaques, the same radiologist manually drew regions of interest (ROIs) around the plaque margins until the ROI contained the full 3D carotid plaque (Figure 1A).

### Feature extraction, selection, and model development

To prevent data heterogeneity and bias, all MRI images were normalized and resampled (2 × 2 × 2 mm) before the radiomics features extraction. Following the guidelines of the Image Biomarker Normalization Initiative (19), this study used Python (version 3.7.0) to import the PyRadiomics (github.com/Radiomics/pyradiomics) toolkit to extract radiomics features, including shape features (2D and 3D), first-order features, gray level co-occurrence matrix (GLCM), gray level size zone matrix (GLSZM), gray level run length matrix (GLRLM), and gray level dependence matrix (GLDM) based original images and Gaussian and wavelet images. After 2 weeks, the same MRI physician randomly selected 20 carotid HRMRIs to re-segment 3D carotid ROIs and extracted radiomics features, and those featuring an interclass correlation coefficient (ICC) of ≥ 0.7 were included in the subsequent study, which was considered to be excellent robustness (20).

The extracted radiomics features typically contained redundancies, many of which were strongly correlated, so the following criteria were used to screen the features in this study. (1) 70 and 30% of the data were randomly divided into training and test sets, respectively. We utilized Z-Score ([value-mean]/standard deviation) distribution to normalize all radiomics features. (2) Univariate analysis was used to select radiomics features with *p* < 0.01. And then, the LASSO (least absolute shrinkage and selection operator) algorithm was used to further reduce the number of features. Using the LASSO algorithm, the most significant features with the smallest deviation were chosen as the final features (Figure 1B). Due to its improved screening of high-dimensional data, the LASSO technique is widely employed in radiomics. (3) In the training cohort, we constructed a multivariate logistic regression radiomics model using the final features, and in the test cohort, we assessed its performance. (4) In this radiomics model, the probability of vulnerability for each patient is determined using a regression-weighted algorithm.

According to previous studies, the traditional radiological models were constructed based on IPH, PE, wall remodeling pattern, and lumen stenosis rates (18, 21). Finally, a combined model was built based on the conventional radiological and radiomics characteristics, and the corresponding nomogram was established by the R software.

### Statistical analysis

All statistical analyses were performed using R 4.2.1 (www.Rproject.org) and python (www.python.org). All continuous variables were reported as mean ± standard deviations (SD), and categorical variables were depicted as count (%). We used the Shapiro-Wilk test to check for normal distribution. To compare clinical characteristics and traditional features between asymptomatic and symptomatic carotid stenosis patients, the student's t test/Mann–Whitney *U*-test was used for quantitative variables, and the chi-square test/Fisher's exact test was used for categorical variables. Univariate logistic regression and the LASSO algorithm were performed for the final screening of radiomics characteristics. Receiver operating characteristic (ROC) calculations were made for each model to evaluate the identification of vulnerable plaques. The ROCs were compared using Delong testing. The statistical significance was determined by the two-tailed *p*-value of < 0.05.

## Results

### Patient characteristics

Ninety patients with carotid stenosis who underwent HRMRI were included, 48 (53.33%) had MRI findings suggestive of acute phase cerebral infarction ipsilateral to the carotid stenosis. There were no significant differences between asymptomatic and symptomatic carotid stenosis patients in terms of gender, age, BMI, history, medication history, and lipids (Table 1). A median of 8.0 days passed between an HRMRI and an ischemic cerebrovascular incident (interquartile range: 4.0–13.75 days).

### Radiomics assessment of the carotid plaque

On both raw and filtered pictures, 4,170 features were initially collected from each ROI. In terms of the intraclass correlation coefficient, 1,563 characteristics (37.5%) showed outstanding robustness. After univariate analysis and LASSO feature screening, 13 features, including 5 features on 3D T_1_-SPACE and 8 features on 3D T_1_-SPACE-CE sequence, were ultimately chosen and used to create the multifactorial logistic radiomics model. The formulation of the radiomics signature is as Figure 1C.

**Figure 2:**
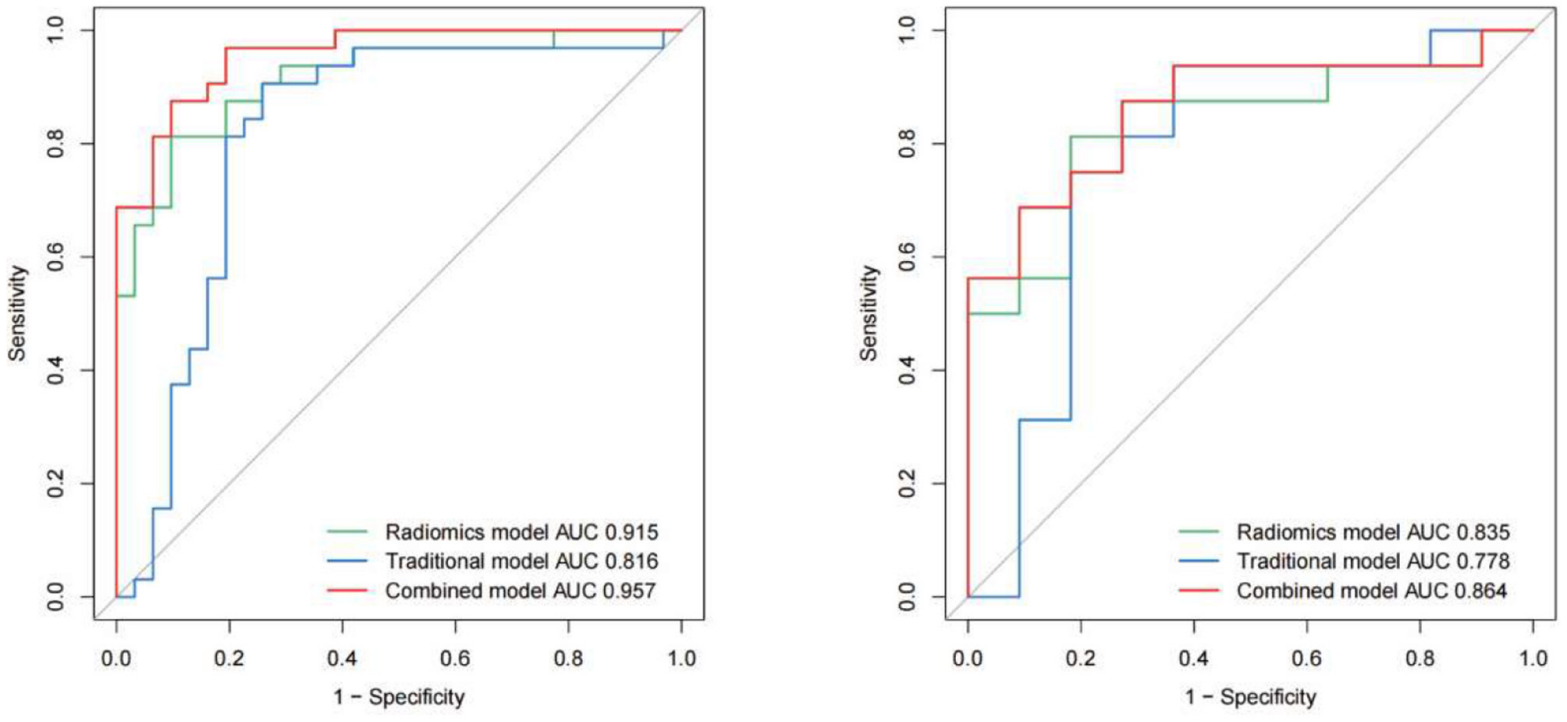
Receiver operating characteristic (ROC) curves of all the models (traditional model, radiomics model and combined model) in the training and test cohorts respectively.

Five-fold cross-validation was used to simulate the discriminatory power of the 3D carotid radiomics model, which had AUC values of 0.915 (95% CI: 0.85–0.98) for the training set and 0.835 (95% CI: 0.68–0.99) for the testing set. And it had the desirable discrimination ability with a specificity of 63.6% and a sensitivity of 93.8% in the testing set (Table 2, Figure 2).

**Table 2:** Performance of the models in training and testing cohorts.

**Assessment models**	**Cohort**	**AUC (95% CI)**	**Sensitivity**	**Specificity**
Radiomics model	Training	0.915 (0.846–0.984)	0.812	0.903
	Testing	0.835 (0.677–0.993)	0.938	0.636
Traditional model	Training	0.816 (0.697–0.934)	0.906	0.742
	Testing	0.778 (0.567–0.990)	0.812	0.818
Combined model	Training	0.957 (0.915–0.999)	0.875	0.903
	Testing	0.864 (0.721–1.000)	0.875	0.727

### Traditional and combined assessment models

**Figure 3:**
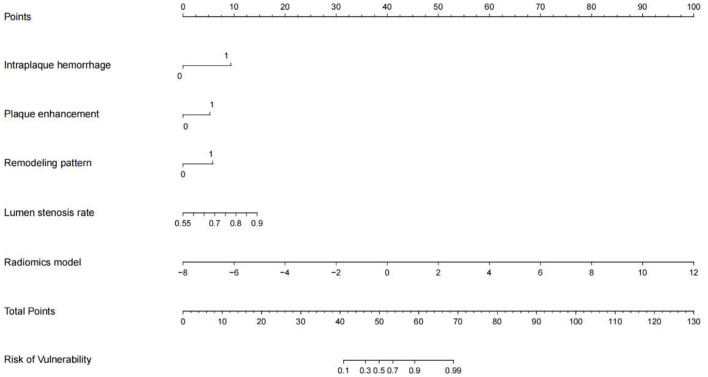
A nomogram integrating the radiomics scores and traditional features of the training sets.

IPH and luminal stenosis rate were found to be linked with symptomatic plaques according to the univariate analysis results (*p* < 0.05, Table 2). Although there were no significant differences in PE and wall remodeling patterns between the two groups, we created models employing IPH, PE, wall remodeling pattern, and lumen stenosis to improve the performance of the traditional model. With a sensitivity of 90.6% and a specificity of 74.2%, the traditional model produced an AUC of 0.816 (95% CI: 0.70–0.93) for the training set and an AUC of 0.778 (95% CI: 0.57–0.99) for the testing set. The final combined model has an AUC in the training set of 0.957 (95% CI: 0.92–1.00) and the testing set of 0.864 (95% CI: 0.72–1.00) (Table 2, Figure 2). The related nomograms for estimating the risk of ischemic cerebrovascular episodes are depicted in Figure 3.

The DeLong test revealed that the ROC curves of the combined model performed better than those of the traditional model in both the training and testing groups (*p* = 0.005 and *p* = 0.037, respectively). However, in both cohorts, there was no significant difference between the combined model and the radiomics model (*p* > 0.05). The calibration curve reveals a sufficient correlation between the diagnostic results of our radiomics and combined model and the actual results in the two sets (Figure 1Cb, c). The decision curves showed that, in the range of 0 to 1, decisions based on our radiomics and combined model achieved a net benefit over “no treatment” or “all treatment” (Figure 1Cd, e).

## Discussion

In patients with carotid stenosis, high-risk plaque poses a significant risk of developing cerebrovascular embolic events. Numerous carotid plaque MRI investigations have been carried out to explore plaque components or characteristics linked to cerebral ischemic episodes. However, most of the early studies have emphasized only conventional qualitative assessments (8). In this study, we constructed a radiomics model to identify vulnerable carotid plaques by extracting 3D carotid plaque radiomics features from HRMRI using a radiomics technique. Excellent diagnostic performance was demonstrated in identifying vulnerable carotid plaques, allowing quantitative scoring of each plaque's vulnerability to indicate the risk of stroke.

To the authors' knowledge and literature review, this study is the first study to develop a 3D carotid plaque radiomics diagnostic model for identifying vulnerable carotid plaques based on 3D T_1_-SPACE. Previous research has only extracted radiomics features at the MRI level, where the plaque area is the biggest. However, the generalizability of the previous model is diminished due to the model's extensive radiomics content. In addition, although the previous combined model outperforms our model, its AUCs per sequence were 0.846, 0.826, 0.857, and 0.816, which were inferior to our radiomics model (AUC of 0.915) (13). Compared to single-layer carotid plaques, 3D plaques can more accurately indicate plaque vulnerability and rupture risk. Arna et al. used ultrasonography to create a model of 3D plaque ultrasound texture paired with plaque volume, which was considerably more accurate at predicting future cerebrovascular events than the conventional risk classification techniques (22). Saba et al. discovered that with an increase in plaque volume, lipid levels and proportion of calcification also increase, in addition to a correlation between the volume of lipid component and plaque surface ulceration, and is a significant risk factor for cerebrovascular events (23). Cai et al. studied 63 patients for up to 55.1 months and discovered that advancement in carotid plaque volume was independently linked to recurrent ischemic cerebrovascular episodes (24). Therefore, feature extraction and evaluation of 3D carotid plaques may enhance the diagnostic performance of vulnerable plaques.

The latest guidelines published by the European Society of Vascular Surgery currently recommend optimal medication treatment for asymptomatic patients with 60% stenosis, favor revascularization for patients at average surgical risk with 60–99% stenosis, and affirm the importance of radiological evaluation in decision-making (25). However, a meta-analysis by Joseph et al. revealed that about 26.5% of patients with asymptomatic carotid stenosis had coupled vulnerable plaques, and this correlated with a greater incidence of ipsilateral ischemic cerebrovascular episodes. However, the prevalence of high-risk plaques was unrelated to the degree of stenosis (26). Therefore, regardless of the level of stenosis, it is essential to identify plaques at risk of causing cerebrovascular events using radiological techniques. Previous studies have shown that the HRMRI features of symptomatic plaque mainly include IPH, PE, wall remodeling pattern, and lumen stenosis. However, it only allows for qualitative judgments and requires a specialist with radiological expertise and substantial work experience. Similar to previous findings in coronary and cerebral plaque studies, we discovered that the radiomics model outperformed the conventional model in diagnosing plaque state (AUC = 0.915 vs. 0.816) (14, 27). What's more, optimal diagnostic performance can be achieved when conventional radiological and radiomics features are combined. The decision curves depicted demonstrate that the combined model caused a net benefit for patients in both the training and testing sets. Therefore, the nomogram developed in this study is a useful tool in clinical practice (Figure 3).

However, there are some limitations to this study. Our study had a limited sample size. Although cross-validation was performed to enhance our model's performance, it may still be susceptible to over- or under-fitting. In addition, using single-center data analysis, the same scanning instrument, and set MRI parameters might have limited the generalizability of the model. Also, the ROI could be manually segmented before the extraction of radiomics features. Although the hand-drawn method is regarded as the “gold standard” for image segmentation now, the process is tedious and time-consuming. Despite rapid advancements in deep learning semantic segmentation algorithms for automatic segmentation of ROIs in recent years, its clinical use requires the development of accurate and interpretable algorithmic codes. Thus, future studies might integrate multi-omics techniques that would include clinical data, radiomics, gene proteomics, and hemodynamics to further enhance the prediction of cerebrovascular ischemia risk in patients with carotid stenosis.

## Conclusion

HRMRI-based 3D carotid radiology models can improve the performance of traditional radiology in identifying vulnerable carotid plaques. One major advantage of radiomics analysis is its ability to extract quantitative data from images which enhances the diagnostic performance beyond traditional evaluation. Future prospective studies could further enhance radiomics in predicting ischemic cerebrovascular episodes in individuals with carotid artery stenosis.

## Data availability statement

The raw data supporting the conclusions of this article will be made available by the authors, without undue reservation.

## Ethics statement

The studies involving human participants were reviewed and approved by the Ethics Committee of First Affiliated Hospital of Zhengzhou University, approval number 2022-KY-0068, and conforms to the ethical guidelines of the 1975 Declaration of Helsinki. The patients/participants provided their written informed consent to participate in this study.

## Author contributions

XZ, ZH, ZJ, RC, and ZL: conception, design, analysis, and interpretation. XZ, ZH, RC, ZJ, JS, and ZL: data collection and statistical analysis. XZ, ZH, CL, and ZL: writing the article. XZ, ZH, ZJ, RC, CL, and ZL: critical revision of the article. XZ, ZH, RC, ZJ, JS, CL, and ZL: final approval of the article. All authors approved the final version to be submitted for publication.
